# Impact of prenatal exposure to maternal stress on infant neurodevelopment

**DOI:** 10.1192/j.eurpsy.2026.11165

**Published:** 2026-07-31

**Authors:** C. Díaz Mayoral, E. A. Sánchez, M. Serrano Molina, J. Gimillo Bonaque

**Affiliations:** 1 Psiquiatría, Hospital Universitario Principe de Asturias; 2 Psiquiatría, Hospital Blua Sanitas Valdebebas, Madrid, Spain

## Abstract

**Introduction:**

Growing scientific evidence underscores the link between maternal stress during pregnancy and future problems in emotional self-regulation, anxiety or ADHD in offspring. Prenatal adversity is recognized as a risk factor for susceptibility to neuropsychiatric illnesses later in life, such as schizophrenia, ADHD and autism spectrum disorders (ASD). The concept of fetal programming postulates that a stimulus during a vulnerable developmental period has a long-lasting or permanent effect, and that its effects depend on the timing of exposure and the developmental stage of organ systems.

**Objectives:**

To review how prenatal exposure to maternal stress impact on infant development and the possible emergence of neurodevelopmental disorders in the child.

**Methods:**

Review of scientific articles on August 12, 2025.

**Results:**

The timing of exposure to maternal stress is crucial, with the first trimester being a period of particular vulnerability for placental epigenetic programming and infant neurodevelopment.

Maternal cortisol and pregnancy-specific anxiety exert independent and programmatic influences on fetal cognitive development, with effects dependent on the timing of exposure.

The placenta is a central organ mediating the transmission of maternal stress to the fetus, showing placental layer-specific epigenetic modifications (chorionic villi vs. maternal decidua) in response to stress signals.

Subtle changes in NR3C1 and FKBP5 methylation in the chorionic villi reflect increased sensitivity to moderate changes in cortisol associated with stress. FKBP5 methylation in maternal decidua is a marker of anticipated labor.

Although maternal depressive symptoms showed no direct association with methylation of the genes studied in a low-risk cohort, more severe pathologies could induce more relevant changes. The low methylation observed in HSD11B2 could indicate an efficient regulation of placental cortisol in the sample, buffering its effects.

**Conclusions:**

These findings reinforce the need for longitudinal assessments with multiple measurements during gestation and underscore the great potential of the placenta as a biomarker of maternal stress and risk for neuropsychiatric and neurodevelopmental disorders.

**Disclosure of Interest:**

None Declared

